# Goat’s Milk Powder Enriched with Red (*Lycium barbarum* L.) and Black (*Lycium ruthenicum* Murray) Goji Berry Extracts: Chemical Characterization, Antioxidant Properties, and Prebiotic Activity

**DOI:** 10.3390/foods14010062

**Published:** 2024-12-29

**Authors:** Danijel D. Milinčić, Aleksandar Ž. Kostić, Steva Lević, Uroš M. Gašić, Dragana D. Božić, Relja Suručić, Tijana D. Ilić, Viktor A. Nedović, Bojana B. Vidović, Mirjana B. Pešić

**Affiliations:** 1 Department of Food Technology and Biochemistry, Faculty of Agriculture, University of Belgrade, Nemanjina 6, 11080 Belgrade, Serbia; danijel.milincic@agrif.bg.ac.rs (D.D.M.); akostic@agrif.bg.ac.rs (A.Ž.K.); slevic@agrif.bg.ac.rs (S.L.); vnedovic@agrif.bg.ac.rs (V.A.N.); 2 Department of Plant Physiology, Institute for Biological Research Siniša Stanković-National Institute of Serbia, University of Belgrade, Bulevar Despota Stefana 142, 11060 Belgrade, Serbia; uros.gasic@ibiss.bg.ac.rs; 3 Department of Microbiology and Immunology, Faculty of Pharmacy, University of Belgrade, Vojvode Stepe 450, 11221 Belgrade, Serbia; dragana.bozic@pharmacy.bg.ac.rs; 4 Department of Pharmacognosy, Faculty of Medicine, University of Banja Luka, 78000 Banja Luka, Bosnia and Herzegovina; relja.surucic@med.unibl.org; 5 Department of Bromatology, Faculty of Pharmacy, University of Belgrade, Vojvode Stepe 450, 11221 Belgrade, Serbia; tijana.ilic@pharmacy.bg.ac.rs

**Keywords:** wolfberry, goat milk-based products, chemical analysis, bioactive compounds, functional additives

## Abstract

The current trend in food innovations includes developing products containing plant ingredients or extracts rich in bioactive compounds. This study aimed to prepare and characterize skimmed thermally treated goat’s milk powders enriched with lyophilized fruit extracts of *Lycium ruthenicum* Murray (GMLR) and *Lycium barbarum* L. (GMLB). Proximate analysis, ultra-high performance liquid chromatography with quadrupole time-of-flight mass spectrometry (UPLC-Q-TOF-MS), Fourier transform infrared spectroscopy using attenuated total reflection (FTIR-ATR), and electrophoretic analysis were assessed. Total phenolic content (TPC), total protein content, and antioxidant properties of enriched goat milk powders were determined spectrophotometrically, and prebiotic potential was evaluated by the broth microdilution method. A total of 25 phenolic compounds and 18 phenylamides were detected in the enriched goat milk powders. Electrophoretic analysis showed the absence of proteolysis in the prepared powders. The GMLR showed the highest TPC and displayed a ferric ion-reducing power, probably contributed by anthocyanins and some phenylamides. GMLR and GMLB had higher ABTS radical scavenging activity but lower ferrous ion-chelating capacity than control goat′s milk powder. GMLB and GMLR in a dose-dependent manner (0.3–5 mg/mL) showed a growth-promoting effect on probiotic strains. In summary, prepared goji/goat milk powders, primarily GMLR, might be used as prebiotic supplements or functional food additives.

## 1. Introduction

Goat milk has been an important part of human nutrition for millennia. However, in recent decades, goat milk has attracted scientific and consumer interest due to its nutritional profile and functional properties [1]. Compared to cow’s milk, goat’s milk is higher in fat, proteins, vitamin A, vitamin B, and calcium contents but lower in lactose [2]. In addition to higher digestibility, goat milk proteins have hypoallergenic potential due to a lower level of αs1-casein than cow milk [3]. Compared to cow’s milk, goat milk proteins have a higher content of some amino acids (tryptophan and cysteine) and oligosaccharides (4–5 times) [3,4], which exert prebiotic, immunomodulatory, and antimicrobial effects and improve intestinal barrier functions [5,6]. Although most production and consumption occurs in Asia, the demand for goat milk products is increasing in Western countries. In addition to direct consumption, raw goat’s milk is used to produce fermented goat dairy products (yogurt, cheese), ice cream, infant formula, and other food products [7].

Due to the increasing knowledge about the bioactive compounds of goat’s milk and its anti-inflammatory, immunomodulatory, anti-hypertensive, anti-diabetic, and bone-strengthening effects, this milk has been recognized as an unexplored food matrix for developing new functional food products and nutraceuticals [8,9]. In addition to nutritional and biological properties [10], good functionality makes goat milk proteins applicable in the formulation of various products [11]. Goat milk proteins could be used as emulsifiers in food products such as salad dressings, mayonnaise, and cream or gelling agents in cheese, yogurt, and puddings. Also, their water-binding properties make them useful for producing meat and plant-based products [10]. Thermally-induced polymerized goat milk whey proteins demonstrated thickening and stabilizing properties, reducing syneresis in yogurt [12]. Furthermore, this by-product of cheese production could be used as material for the microencapsulation of ingredients in fermented product formulations [13]. It has been reported that goat’s milk can be a good carrier of bioactive compounds such as prebiotic substances, including carbohydrates, and other substances like phenolic compounds [4]. In recent years, goat′s milk has been successfully enriched with various sources/extracts rich in phenolic compounds, including medicinal plants [14], *Tinospora cordifolia* (giloy) juice with or without plant extracts [15], monofloral pollen [16], grape pomace seed extracts [17,18,19], and mushroom extracts [20], with the aiming of producing functional food ingredients and/or products. However, goat milk and the production of novel functional goat milk-based products or food supplements with various plant extracts are still insufficiently exploited.

Considering this fact, goji berries (*L. barbarum* and *L. ruthenicum*) can be a promising source of various bioactive compounds for enriching goat’s milk. These fruits present rich sources of highly valuable polysaccharides and “small,” very active compounds such as phenolic compounds, phenylamides, carotenoids, and provitamin C [21,22]. These bioactive compounds have shown various health-promoting effects, including antioxidant protection, immune support, eye health, anti-aging effects, and metabolic regulation [23]. Precisely, previous studies have shown that red (*L. barbarum*) goji berries stimulate the growth of probiotic strains of *Bifidobacterium* and *Lactobacillus* and improve their viability [24,25], suggesting the use of goji berry extracts as prebiotic additives in food and nutraceuticals [25]. In our previous research, we also have demonstrated in vitro prebiotic effects of black (*L. ruthenicum*) goji berries on various probiotics, particularly on the growth of the yeast *Saccharomyces boulardii* [22], primarily due to the high content of anthocyanins [26]. In recent years, the trend of using goji berries in formulating various food products, including dairy products, has been growing [23]. Previous studies reported the effects of adding goji berries or their extracts in fermented dairy products, such as cow′s [24,27,28] and goat’s milk [29] yogurts. Red goji berries (7%) added to yogurt improved the sensory quality, enhanced lactic acid bacteria viability, and increased consumers’ acceptance [24]. Including dried red goji berries in yogurt is proposed as a strategy for producing dairy products with acceptable flavors and antioxidant properties [27]. It has been reported that polyphenol-rich goji berry extracts (0.05, 0.10, and 0.15% (*w*/*v*)) added to yogurt improved the stability of vitamin D during shelf life [28]. Also, it is verified that *L. ruthenicum* fruits (1–2%) added to goat milk yogurt stimulated the proliferation of lactic acid bacteria and improved the goat milk yogurt quality and antioxidant capacity [29].

To our knowledge, the enrichment of goat’s milk powder with goji berry extracts containing phenolics and phenylamides has not yet been carried out. Therefore, this study aimed to prepare and chemically characterize goat milk powder enriched with extracts from black (*L. ruthenicum*) and red (*L. barbarum*) goji berries and analyze their prebiotic and antioxidant activity. Incorporating goji berry extracts into goat milk can be a promising strategy for developing goat-based functional food ingredients.

## 2. Materials and Methods

### 2.1. Preparation of Goji Berry Extracts

Black (*L. ruthenicum*) and red (*L. barbarum*) goji berries were obtained from the private plantation “Ljuba i sinovi” (Niš, southern Serbia). The goji samples were ground and extracted with 80% acidified methanol (1:10 *w*/*v*) for 1 h on a mechanical shaker. After extraction, samples were centrifugated (3000× *g*, for 10 min). Collected supernatants were evaporated to totally remove the solvent, reconstituted in milli-Q water, and lyophilized. The obtained lyophilized black and red goji extracts were used to prepare spray-dried goat’s milk/goji extract powders.

### 2.2. Preparation of Goat′s Milk and Goat′s Milk/Goji Extract Powders

**Figure 1:**
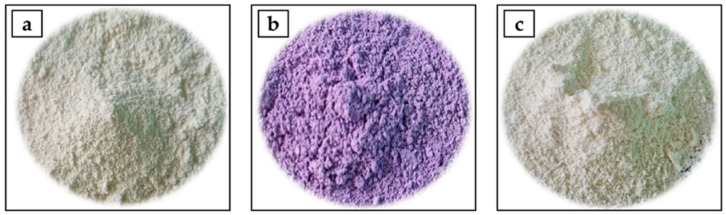
Images of spray-dried powders: (**a**) goat’s milk powder without extract (control sample) (GM); (**b**) goat’s milk powder enriched with *L. ruthenicum* extract (GMLR); and (**c**) goat’s milk powder enriched with *L. barbarum* extract (GMLB).

Before the preparation of powders, goat’s milk was defatted and thermally treated (90 °C, 10 min), as previously described by Pešić et al. [30]. Lyophilized goji extracts (1 g) were mixed with 100 mL of skimmed thermally treated goat’s milk (10 min, on a magnetic stirrer, to obtain a homogeneous mixture). Prepared samples: (1) goat’s milk powder enriched with *L. ruthenicum* extract (GMLR), (2) goat’s milk powder enriched with *L. barbarum* extract (GMLB), and (3) goat’s milk powder without extract (control sample) (Figure 1) were spray-dried with a Buchi Mini B–290 spray dryer (Buchi Labortechnik AG, Flawil, Switzerland), according to the procedure described in detail in our previous study [19]. The obtained powders were packed in cuvettes and stored in a refrigerator for further characterization.

### 2.3. Proximate Analysis

Proximate analysis, including moisture, crude protein and ash content, was performed according to the standard methods [31]. The crude lipid content was estimated according to the protocol described in Ljubobratović et al. [32]. The carbohydrate content was calculated by difference:

Carbohydrates = 100 − (g proteins + g lipids + g ash + g moisture).(1)

### 2.4. FTIR-ATR Spectroscopy of Powders

The prepared goat’s milk and goat’s milk/goji extract powders were recorded by an IRAffinity-1 spectrometer equipped with an ATR unit (Shimadzu, Kyoto, Japan). The spectra were collected in the wavenumber range of 4000–600 cm^−1^, at a resolution of 4 cm^−1^ and from 100 scan accumulations.

### 2.5. UHPLC Q-ToF MS Analysis

To analyze bioactive compounds, goat’s milk and goat’s milk/goji extract powders were extracted with 80% acidified methanol (+0.1% HCl), as previously reported by Milinčić et al. [19]. Briefly, 1 g of powder was extracted with 10 mL of extraction agent for 1 h. After that, samples were centrifuged at 4000× *g* for 10 min. Collected supernatants were filtered through 0.22 µm filters and immediately analyzed by Agilent 1290 Infinity ultra-high-performance liquid chromatography (UHPLC) system coupled with a quadrupole time-of-flight mass spectrometry (6530C Q-ToF-MS) (Agilent Technologies, Inc., Santa Clara, CA, USA). Applied gradient elution program, the flow rate of mobile phases (0.3 mL min^−1^), injection volume of samples (5 µL), operation parameters of ESI source (Dual Agilent Jet Stream electrospray ionization source), and collision energy (30 eV) were the same as previously reported in detail by Milinčić et al. [21]. The chromatographic separation was performed at 40 °C on a Zorbax C18 column (2.1 × 50 mm, 1.8 µm) (Agilent Technologies, Inc., Santa Clara, CA, USA). For screening of prepared extracts (untargeted analysis), Auto MS/MS acquisition mode was used (*m*/*z* = 50–1700 and scan rate 1 spectrum/sec). Agilent MassHunter software (6530C Q-ToF-MS) was used for data evaluation and analysis. Phenylamides and anthocyanins were analyzed in positive ionization mode, while other phenolic compounds were confirmed in negative ionization mode. Accurate masses of components were calculated by using ChemDraw software (version 12.0, CambridgeSoft, Cambridge, MA, USA).

### 2.6. Electrophoretic Analysis of Powders

Protein characterization of prepared powders was carried out by SDS-R-PAGE (sodium dodecyl sulfate–polyacrylamide gel electrophoresis) in reducing conditions, as previously described by Pešić et al. [30] and Milinčić et al. [19]. SDS-R-PAGE was performed using separating (12.5% *w*/*v*; pH = 8.85) and stacking gels (5% *w*/*v*; pH = 6.8), as well as Tris–Glycine running buffer (0.05 M Tris (pH 8.5), 0.19 M glycine, 0.1% *w*/*v* SDS). Briefly, 2 mg of each powder was dissolved in 1 mL of sample buffer containing 5%-mercaptoethanol (0.055 M Tris-HCl (pH 6.8), 2% (*w*/*v*) SDS, 7% (*v*/*v*) glycerol, 0.0025% (*w*/*v*) bromophenol blue, and 5% β-mercaptoethanol). Mixtures were vigorously shaken until complete dissolution of powders, and aliquots of 25 µL were loaded into the wells. After analysis, the gel was stained with Coomassie blue dye for 45 min. Then, the gel was destained, and the scanned gel was analyzed using SigmaGel software (SigmaGel software version 1.1, Jandal Scientific, San Rafael, CA, USA).

### 2.7. Total Phenolics, Total Proteins, and Antioxidant Properties of Powders

Goat’s milk and goat’s milk/goji extract powders (1 g) were reconstituted in 100 mL of milli-Q water, intensively mixed and used for spectrophotometric characterization, including total phenolic content (Folin–Ciocalteu method), total protein content (Bradford method) and antioxidant activities (ferric ion-reducing power (FRP), ABTS^•+^ radical scavenging activity (ABTS^•+^), and ferrous ion-chelating capacity (FCC)). All applied assays were previously detailed and described in research articles [16,19,33,34].

### 2.8. Prebiotic Activity of Powders

The prebiotic activity of goat′s milk and goat′s milk/goji extract powders was tested on four probiotic strains of Gram-positive bacteria (*Lactobacillus plantarum* Lp 299v, *Lactobacillus reuteri* Protectis (DSM 17938), *Lactobacillus rhamnosus* GG (LGG) and *Streptococcus salivarius* subsp. *thermophilus* ST-21), a yeast strain *Saccharomyces boulardii*, and a mixture of the probiotic bacteria *Lactobacillus helveticus*, *Lactobacillus rhamnosus*, and *Bifidobacterium longum*. The microorganisms were prepared under standard conditions, and the prebiotic assays were performed as previously described by Ilić et al. [22]. The powders were tested in the 0.312–5 mg/mL range. The results were calculated as the percentage of growth stimulation of the probiotic strains cultured with the prepared powders compared to the growth of the positive controls (i.e., the growth of the probiotic strains without the addition of prepared powders) according to the following formula:

(2) $$\%\;of\;prebiotic\;effect=\frac{ODsample}{ODpositive\;control}\times 100.$$

### 2.9. Statistical Analysis

All analyses were conducted in triplicate. The results were presented as mean values ± standard deviation (SD). Significant differences between the means of samples were determined using Tukey’s test at *p* < 0.05 (StatSoft Co., Tulsa, OK, USA).

## 3. Results and Discussion

### 3.1. Proximate Composition 

The results of the proximate analysis of goat′s milk and goat′s milk/goji extract powders are shown in Table 1.

Including goji berry extracts in skimmed thermally treated goat’s milk significantly increased ash and fat content (*p* < 0.05). Different moisture content among analyzed samples could explain the variation in proximate composition between goat′s milk and goji/goat’s milk powders.

### 3.2. FTIR-ATR Characterization of Powders

**Figure 2:**
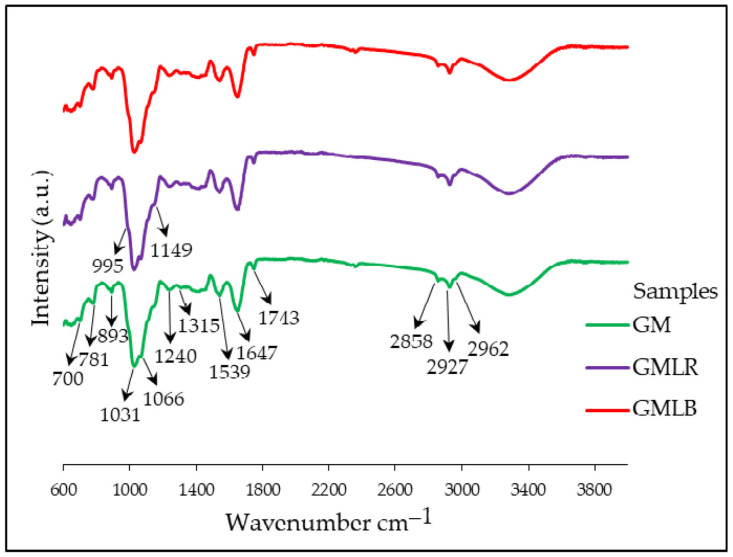
FTIR-ATR spectra of goat′s milk (GM) and goat′s milk/goji extracts (GMLR and GMLB) powders. Abbreviations: “GMLR”—goat’s milk powder enriched with *L. ruthenicum* extract; “GMLB”—goat’s milk powder enriched with *L. barbarum* extract; and “GM”—goat’s milk powder without extract (control sample).

Goat′s milk and goat′s milk/goji extract powders were analyzed using FTIR-ATR to investigate possible interactions between goat′s milk and goji extract compounds. In recorded FTIR-ATR spectra (Figure 2), broad bands with intense peaks can be observed in the carbohydrate (1000–1200 cm^−1^) and protein (1500–1600 cm^−1^ and 1600–1700 cm^−1^) regions. The spectra obtained are similar to the previously published FTIR-ATR spectra of ultra-high-temperature-treated skim cow’s milk [35,36] and thermally treated skim goat′s milk [16,17]. Broad absorption bands with peaks at 1539 and 1640 cm^−1^ corresponded to amide I (ν(C=O) vibration of the peptide bond) and amide II (δ(N–H) and ν(C–N) vibrations), respectively [16,17,35]. The spectral region of 1200–1500 cm^−1^ belongs to amide III, carbohydrates, phenolics, and/or lipids. However, observed low-intensity peaks in this region (1240 and 1315 cm^−1^) and a peak at 700 cm^−1^ probably originated from C–H and/or N–H vibrations of amide III [37]. A peak at 995 cm^−1^ was assigned to vibrations of phosphate groups (-PO32-), which are part of milk proteins [17]. The spectral region of 1030–1150 cm^−1^ and peaks in this region (1031, 1066, and 1149 cm^−1^) were attributed to lactose (C-O vibrations) [37]. Peaks at 781 and 893 cm^−1^ can be related to vibrational modes of C–OH groups present in the structure of saccharides (lactose) [16]. Low-intensity peaks at 2858, 2927, and 2962 cm^−1^ can be related to the C–H stretching vibration of carbohydrates (disaccharides) or residual lipids [37,38]. The peak at 1743 cm^−1^ with C=O stretching vibrations originates from amino acids, lipids, or esters [35]. Finally, the spectra of powders of goat′s milk/goji extracts (GMLR and GMLB) are the same as the spectra of control goat’s milk, with the same bands mainly belonging to milk protein and lactose. No bands originating from goji extracts or shifts indicating interactions between milk proteins and goji phenolics/phenylamides can be observed in the goat’s milk/goji extract powder spectra.

### 3.3. UHPLC Q-ToF MS Characterization of Phenolic Compounds in Methanol Extracts of Powders

As shown in Table 2, untargeted UHPLC Q-ToF MS analysis of the methanolic extracts of powders revealed differences/similarities in the profiles of phenolic compounds among samples. A total of 25 phenolic compounds were identified, mainly phenolic acid derivatives and flavonoid glycosides. As expected, with the exception of dihydroxybenzoic acid, other phenolic compounds were not detected in the control goat’s milk powder (GM). In view of this fact, all phenolic compounds detected in the GMLR and GMLB samples originate from goji berry extracts and directly influence the functional properties of these powders. Hydroxybenzoic acid and dihydroxybenzoic acid were detected in both powders, while hexosides of hydroxybenzoic acid (two isomers) were only confirmed in GMLR powder. Derivatives of hydroxycinnamic acid (coumaric acid, caffeic acid, and ferulic acid) have been identified mainly in the form of esters with quinic acid and/or as glycosides. Coumaric acid hexoside and coumaric acid rhamnosyl hexoside (compound **9**, Table 2) were only found in the GMLR powder, while coumaric acid dihexoside was confirmed in both powders enriched with goji extracts. Compound **9** was recognized as coumaric acid rhamnosyl hexoside (*m*/*z* 471) based on typical fragments at [163 *m*/*z* (coumaroyl moiety)-119 *m*/*z* (-CO_2_)] and 309 *m*/*z* (obtained by the loss of the hexosyl unit). Caffeic acid derivatives were found to be the most numerous and abundant in both powders enriched with goji extracts, which is consistent with previous characterizations showing a dominant presence of these compounds in various goji extracts [21,39,40,41]. Different forms of caffeic acid derivatives were identified in GMLR, such as caffeic acid hexoside, di-caffeoylquinic acid (*m*/*z* 515.1226), and caffeoylquinic acid and its hexosides (*m*/*z* 515.1414) (two isomers). On the other hand, only isomers of caffeoylquinic acid hexosides were identified in GMLB. Ferulic acid and its dihexoside were only confirmed in GM powder enriched with *L. barbarum* extract. The presence of ferulic acid dihexoside and its high content in *L. barbarum* fruit has been reported in other studies [21,42]. All detected flavonoids were differently distributed in GMLR and GMLB powders and depended on the goji extracts used for their preparation. Rutinoside (or [(6“-*O*-rhamnosyl)-hexoside]) of kaempferol, quercetin, and isorhamnetin were detected in GMLB powder. These flavonol glycosides were previously found and described as typical flavonoids of *L. barbarum* [21,40,42,43]. On the other hand, naringenin glycosides such as prunin and naringin, and one laricitrin derivative (compound **22**, Table 2) were identified in GMLR powder. Naringenin glycosides have already been detected and reported in *L. ruthenicum* Murray, especially naringin [44,45,46]. Anthocyanins were only detected in GMLR powder, which give the powder its specific color (Figure 1b) and contribute significantly to the functionality of this powder. All anthocyanins detected were petunidin derivatives of high molecular weight (compounds **23**–**25**, Table 2). These petunidin and other anthocyanidin derivatives from *L. ruthenicum* have been extensively studied over the last decade [22,39,47,48,49,50]. These compounds have characteristic structures that were previously confirmed by NMR [48] and typical MS fragmentation in the positive ionization mode (Table 2).

### 3.4. UHPLC Q-ToF MS Profile of Lycium Phenylamides in Methanolic Extracts of Powders

**Figure 3:**
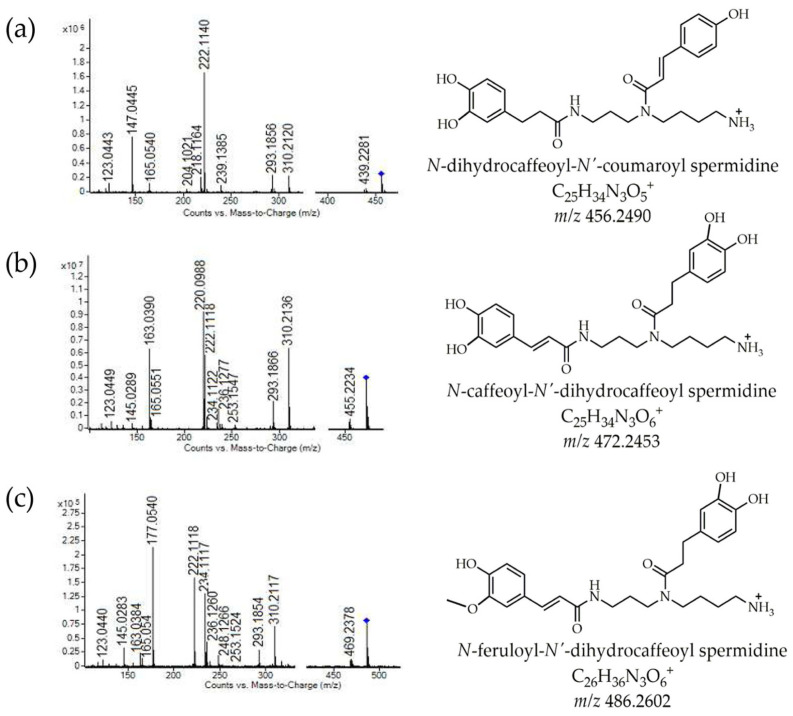
Characteristic MS/MS fragmentation patterns and predicted structures of non-glycosylated spermidine derivatives: (**a**) *N*-dihydrocaffeoyl-*N*′-coumaroyl spermidine (*m*/*z* 456); (**b**) *N*-caffeoyl-*N*′-dihydrocaffeoyl spermidine (*m*/*z* 472); and (**c**) *N*-feruloyl-*N*′-dihydrocaffeoyl spermidine (*m*/*z* 486).

Goji berry is a rich source of phenylamides, which justifies the presence of these bioactive compounds in GM powders containing goji extracts. As expected, no phenylamides were detected in the control GM powder. The individual phenylamides confirmed in the methanolic extracts of the powders are listed in Table 3. Non-glycosylated and glycosylated spermidine derivatives were the most numerous and most frequently detected, followed by spermine and putrescine derivatives. As can be seen, the presence of individual phenylamides in GMLR and GMLB powders depends on the varieties of goji berries used in the powder formulations (Table 3). The phenylamides detected were more numerous in GMLR powder than in GMLB, especially spermidine derivatives. Caffeoyl putrescine (*m*/*z* 251) was found in GMLR powder, while its glycoside (monohexoside) (*m*/*z* 413) was detected in GMLB. The presence of glycosylated caffeoyl putrescine in *L. barbarum* and the proposed fragmentation pathway have been reported in our previous study [21]. Of the identified non-glycosylated spermidines, N,N′-bis-dihydrocaffeoyl spermidine and N-caffeoyl-N′-dihydrocaffeoyl spermidine were confirmed in both GM powder containing goji extracts, while other phenylamides from this class were only found in GMLR (compounds **28**, **29**, and **32**, Table 3). Caffeoyl and/or dihydrocaffeoyl spermidines are typical derivatives of spermidine, which have been found most frequently so far and reported for various goji berries [21,51,52,53,54]. Characteristic MS/MS fragmentation patterns and proposed structures of phenylamides 28 (*m*/*z* 456), 30 (*m*/*z* 472), and 32 (*m*/*z* 486), with the major fragments are shown in Figure 3. Glycosylated spermidines, various mono- and dihexosides (glucosides) of caffeoyl and/or dihydrocaffeoyl spermidines (compounds from **33** to **37**, Table 3), were the most commonly found phenylamides in both (GMLR and GMLB) powders. These glycosylated forms of phenylamides are typical and recently identified compounds in goji berry [21,51,52,53,54]. Trihexoside of caffeoyl/dihydrocaffeoyl spermidine (compound **38**, Table 3) was confirmed only in GMLB, which is consistent with previous studies identifying and describing these compounds (spermidines with three or more sugar units) in *L. barbarum* [21,51,52]. In addition to the spermidine derivatives, various non-glycosylated and glycosylated caffeoyl and/or dihydrocaffeoyl spermine derivatives (compounds from **39** to **43**, Table 3) were confirmed in the analyzed powders with goji extracts. Non-glycosylated caffeoyl and/or dihydrocaffeoyl spermine (compounds such as kukoamines) were only identified in GMLR. Other authors have already detected and analyzed these compounds in *L. ruthenicum* [54,55]. Glycosylated spermine derivatives have only recently been characterized in *L. barabarum* [21,52]. However, in this study, glycosylated caffeoyl and/or dihydrocaffeoyl spermine derivatives (monohexoside spermine derivatives) were also identified and described in GMLR, and they represent new compounds confirmed in *L. ruthenicum*. Glycosylated spermines were identified based on typical MS/MS fragments (Table 3) obtained by the loss of sugar units (sequential loss of sugar units) and cleavage of the polyamine core, as previously reported [21,52]. Interestingly, monohexoside spermine derivatives were found only in GMLR, whereas dihexoside spermine derivative was identified only in GMLB. This could indicate differences in the goji berry profiles, reflected in the differences between the prepared powders of GMLR and GMLB.

### 3.5. Electrophoretic Analysis of GM, GMLR, and GMLB Powders

**Figure 4:**
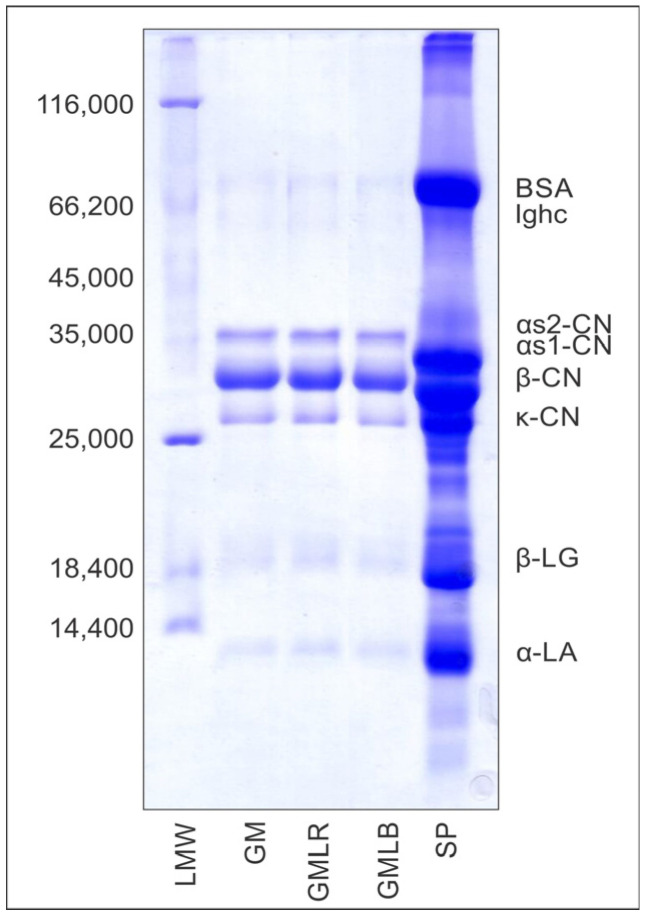
Electrophoretic patterns of goat′s milk (GM) and goat′s milk/goji extract (GMLR and GMLB) powders, analyzed by SDS-PAGE in reducing conditions. Lines: “GMLR”—goat’s milk powder enriched with *L. ruthenicum* extract; “GMLB”—goat’s milk powder enriched with *L. barbarum* extract; “GM”—goat’s milk powder without extract (control sample); molecular weight standard (LMW); and bovine milk protein standard (SP). Abbreviations: bovine serum albumin (BSA); immunoglobulin hard chain (Ighc); αs2-casein (αs2-CN); αs1-casein (αs1-CN); β-casein (β-CN); κ-casein (κ-CN); β-lactoglobulins (β-LG); and α-lactalbumin (α-LA).

The electrophoretic patterns of GM, GMLR and GMLB under reducing conditions are shown in Figure 4. All three samples showed the presence of six dominant goat’s milk protein bands corresponding to α_S_-, β-, and κ-casein and serum proteins [19,30]. The addition of goji berry extracts did not alter the protein composition of the prepared powder, which is similar to the results reported for goat’s milk powder fortified with grape pomace seed extracts [19] but different from the results obtained for goat’s milk powder enriched with *Agaricus blazei Murrill ss Heinem* extract [20]. This suggests that goji extracts do not contain enzymes with proteolytic activity, which is the case with mushroom extracts. On the other hand, goat milk proteins can be good carriers for bioactive compounds from the fruits of *L. barbarum* and *L. ruthenicum*, similar to previously reported for phenolics from grape pomace seeds [19] and biocompounds from various medicinal plants [14,15].

### 3.6. Total Phenolic, Total Protein Content, and Antioxidant Properties

The total phenolic content, total protein content, and antioxidant properties of the prepared powders are shown in Table 4. TPC was the highest in GMLR, followed by GM, and the lowest value was recorded in the GMLB sample. The TPC value for GMLR, which is higher than for GMLB, is in accordance with the phenolic profile of powders presented in Table 1. GMLR exhibited a greater diversity of phenolic compounds compared to GMLB. The greatest variation was noted in the anthocyanins, which was detected only in the GMLR sample. Although the GM sample contained only the dihydroxybenzoic acid as a phenolic compound, the TPC value of GM is high due to the presence of other compounds, such as proteins and lactose, that can react with the Folin–Ciocalteu reagent, resulting in overestimated TPC values [19]. The results presented in Table 4 show that GM with 35.44 mg BSA/100 mL had the highest TPrC level compared to GMLB and GMLR, whose protein content was not statistically different.

Phenolic compounds play a crucial role in the antioxidant potential of plant extracts by donating hydrogen atoms or electrons to neutralize free radicals, stop chain reactions triggered by oxidative stress, and protect cell membranes from damage. They can also bind to metal ions such as iron and copper and prevent them from catalyzing the formation of reactive oxygen species (ROS) through Fenton and Haber–Weiss reactions. Certain phenolic compounds inhibit oxidative enzymes such as lipoxygenase and xanthine oxidase and thus reduce the production of ROS [56]. Three antioxidant assays were used in this study to comprehensively analyze and understand the extracts tested, reflecting the antioxidant activity of prepared powders. The FRP assay showed that only the GMLR sample exhibited a ferric ion-reducing capacity of 13.98 µg AA/mL. The ferric-reducing capacity of this sample (GMLR) can be due to phenolic compounds, primarily anthocyanins, which can act as free radical scavengers and potent reducing agents [57]. In addition to phenolic compounds, GMLR powder contains various phenylamides (Table 3), which also contribute to the ferrous-reducing capacity of this powder. They have been extensively studied for their beneficial health effects, including their antioxidant potential [58]. The ABTS^•+^ assay demonstrated that all samples could neutralize free radicals by releasing hydrogen atoms or electrons from various compounds. However, GM with goji extracts (primarily GMLR) possesses better scavenging activity against ABTS^•+^ free radicals, significantly higher than control GM. The ability of GMLR and GMLB powders to scavenge free radicals was probably the most contributed by phenolic compounds and phenylamides [19]. Control goat′s milk also showed ABTS^•+^ radical scavenging activity, primarily contributed by milk proteins [19,59]. All samples showed ferrous chelating properties due to high protein content, as shown by electrophoretic analysis. Control goat milk showed the best chelating properties probably due to the highest content of natural chelators, such as casein and lactoferrin, which can bind iron [60]. On the other hand, the addition of goji extract to goat’s milk significantly decreased the chelating properties of both GMLR and GMLB powders. However, these GM powders with goji extracts had better chelating ability than goat’s milk powder enriched with grape pomace seed extracts [19]. The reduction in the chelating capacity of GMLR and GMLB powders may be due to the presence of WP/CN complexes on the surface of casein micelles and their interactions with phenolic compounds and phenylamides [19,61,62], resulting in a reduced ability of goat milk proteins to bind ferrous ions.

### 3.7. Prebiotic Activity of Powders

The importance of phenolic compounds in modulating the gut microbiota as novel potential prebiotics has been extensively studied and well-reviewed over the last decade [63,64]. Probiotic strains of the Gram-positive bacteria *Lactobacillus plantarum*, *Lactobacillus reuteri*, *Lactobacillus rhamnosus*, *Streptococcus thermophilus*, and a mixture of *Lactobacillus and Bifidobacterium* possess well-documented health benefits and ability to enhance immune response, inhibit pathogenic bacteria, and improve the gut microbiota [65]. These strains have already been used in numerous clinical studies to treat various diseases [66,67,68]. In addition, these strains are commercially available as over-the-counter probiotic supplements. Combining these probiotic strains with goji berry extracts and goat milk powder aims to create a synergistic effect, potentially boosting the final product’s prebiotic activity and nutritional value [67]. Previous studies have shown good prebiotic potential of various phenolic compounds from berries in promoting the growth of beneficial bacteria and suppressing the proliferation of pathogenic bacteria, thus maintaining the host’s gut homeostasis [64]. The concentration range of 0.312–5 mg/mL for testing the prebiotic potential of goat milk powder-enriched red and black goji berry extracts was chosen to cover a broad spectrum of possible effects on probiotic bacteria. This spectrum allows researchers to observe the dose-dependent responses of probiotic bacteria to the extracts and determine the optimal concentration for maximum prebiotic effect. At lower concentrations (0.312–1 mg/mL), the extracts can exhibit mild prebiotic effects and promote the growth of beneficial bacteria without overwhelming the system. Higher concentrations (2–5 mg/mL) are tested to observe more pronounced effects and to determine the threshold at which the extracts may have an optimal prebiotic effect. A similar concentration range is commonly used in studies on the prebiotic potential of goji berries [22,25,69], e.g., 0.312–5 mg/mL [22] or 2.5%, 5%, 10%, and 15% (*w*/*v*) [69]. Higher concentrations (10 to >20 mg/mL), on the other hand, could have an inhibitory effect on probiotic bacterial strains, as they also have on pathogenic strains [70].

**Figure 5:**
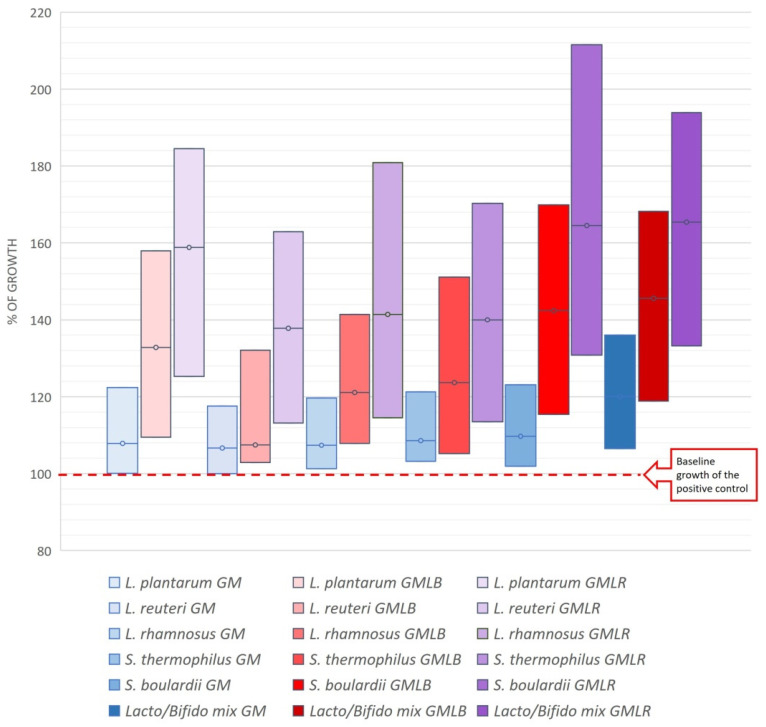
The prebiotic potential of the goat′s milk and goat′s milk/goji extract powders on various probiotic strains of microorganisms. The results are presented as minimum and maximum percent of growth stimulation of prepared powders in the range of 0.312–5 mg/mL. Abbreviations: “GMLR”—goat’s milk powder enriched with *L. ruthenicum* extract; “GMLB”—goat’s milk powder enriched with *L. barbarum* extract; and “GM”—goat’s milk powder without extract (control sample).

The results of the prebiotic potential of goat’s milk and goat’s milk/goji extract powders are shown in Figure 5 and Table 5. The stimulation of the growth of the probiotic strain was dose-dependent and recognizable at all applied concentrations of prepared powders. Both tested goat’s milk/goji extract powders (GMLR and GMLB) had better and statistically significant effects on the stimulation of the growth of the probiotic strain compared to the control goat’s milk. This means that the goji compounds contained in the powders contributed to the improvement of the prebiotic activity of the prepared goat’s milk powders with goji extracts. These results are supported by recent studies showing good prebiotic properties of *L. ruthenicum* extract [22] and encapsulated powder of *L. barbarum* in stimulating the growth and viability of probiotic *Bifidobacterium* and *Lactobacillus* strains [25]. Control goat′s milk also showed prebiotic activity, primarily contributed by goat milk oligosaccharides [6]. Further, the GMLR powder had a more pronounced effect on growth stimulation of all tested probiotic strains compared to the GMLB (*p* < 0.05). Differences in the prebiotic activity of these powders are obviously due to differences in their profile of bioactive compounds, primarily in the presence of anthocyanins, as shown by UHPLC Q-ToF MS analysis. Previously, studies have shown the positive effects of *L. ruthenicum* anthocyanins on the regulation of gut microbiota, contributed by the prevention of induced obesity [71] and inflammatory disease [26] caused by a high-fat diet. Finally, tested goat′s milk/goji extract powders had the most pronounced growth-promoting effect on *Saccharomyces boulardii* and the *Lactobacillus*/*Bifidobacterium* mixture of the probiotic strains. These results are in agreement with our previous findings [22], which showed high prebiotic activity of *L. ruthenicum* extracts for various bacteria and yeasts, especially for *Saccharomyces boulardii*.

## 4. Conclusions

This study investigated the chemical characterization, antioxidant properties, and prebiotic activity of thermally treated skimmed goat′s milk powder enriched with red (*L. barbarum*) and black (*L. ruthenicum*) goji berry extracts. A total of 25 phenolic compounds and 18 phenylamides and their derivatives were detected in the prepared samples but with different frequencies depending on the goji berry species. Phenolic acid derivatives and flavonoid glycosides were present in both preparations. In contrast, anthocyanins were only detected in the preparation with black goji berry extract, which gave the powder a specific color and functionality. Phenylamides were also present in these powders, with non-glycosylated and glycosylated spermidine derivatives predominating, followed by spermine and putrescine derivatives. Adding lyophilized goji berry extracts to goat milk did not affect its protein composition, as confirmed by electrophoretic analysis. Enrichment of thermally treated skimmed goat milk with both goji berries extracts significantly increased radical scavenging activity. In addition, based on the growth-promoting effects of prepared powders on *Saccharomyces boulardii* and the *Lactobacillus/Bifidobacterium* mixture of probiotic strains, they could be considered prebiotic supplements or functional food additives.

## Figures and Tables

**Table 1:** Proximate composition of goat′s milk and goat′s milk/goji extract powders (%).

Parameter	GMLR	GMLB	GM
Moisture	6.10 ± 0.12 ^b^	8.99 ± 0.15 ^a^	4.52 ± 0.11 ^c^
Ash	9.04 ± 0.45 ^a^	8.2 ± 0.48 ^b^	6.85 ± 0.55 ^c^
Protein	32.16 ± 0.35 ^a^	31.71 ± 0.41 ^a^	31.71 ± 0.43 ^a^
Fat	4.02 ± 0.11 ^a^	4.61 ± 0.12 ^a^	3.63 ± 0.10 ^b^
Carbohydrate	48.68 ± 0.36 ^b^	46.49 ± 0.45 ^b^	53.29 ± 0.40 ^a^

Abbreviations: “GMLR”—goat’s milk powder enriched with *L. ruthenicum* Murray extract; “GMLB”—goat’s milk powder enriched with *L. barbarum* L. extract; and “GM”—goat’s milk powder without extract (control sample). Values are presented as mean ± standard deviation (mean ± SD). Different letters in the same row denote significant differences (*p* < 0.05), according to *Tukey’s* test.

**Table 2:** UHPLC Q-ToF MS identification and characterization of phenolic compounds in methanolic extracts of GM, GMLR, and GMLB powders. Target compounds, expected retention time (RT), molecular formula, calculated mass, exact mass, and MS2 fragments are presented in Table 2.

No	RT	Compounds	Formulas	Calculated Mass	*m*/*z* Exact Mass	mDa	MS Fragments (Main Fragment)	Samples		
								GMLR	GMLB	GM
***Phenolic acid and derivatives***										
1	4.29	**Hydroxybenzoic acid**	C_7_H_5_O_3_^–^	137.0239	137.0249	1.03	**108.0218(100)**	**+**	**+**	**−**
2	5.64	**Dihydroxybenzoic acid (like gentisic acid)**	C_7_H_5_O_4_^–^	153.0188	153.0206	1.82	**109.0293(100)**, 135.0069	**+**	**+**	**+**
3	7.97	**Ferulic acid**	C_10_H_9_O_4_^–^	193.0501	193.0512	1.12	**134.0357(100)**, 133.0342, 148.9154, 178.0351	**−**	**+**	**−**
4	2.61	**Hydroxybenzoic acid hexoside is. I**	C_13_H_15_O_8_^–^	299.0767	299.0794	2.71	**137.0241(100)**	**+**	**−**	**−**
5	3.24	**Hydroxybenzoic acid hexoside is. II**	C_13_H_15_O_8_^–^	299.0767	299.0794	2.71	**137.0246(100)**	**+**	**−**	**−**
6	6.40	**Coumaric acid hexoside**	C_15_H_17_O_8_^–^	325.0923	325.0947	2.36	**145.031(100)**, 119.0501, **163.0328**	**+**	**−**	**−**
7	5.41	**Caffeic acid hexoside**	C_15_H_17_O_9_^–^	341.0873	341.0925	5.24	**161.0241(100)**, 133.0291, 135.0443, **179.0358**	**+**	**−**	**−**
8	6.31	**Caffeoylquinic acid (like chlorogenic acid)**	C_16_H_17_O_9_^–^	353.0873	353.0900	2.74	**191.0558(100)**, 127.0401, 161.0242, 173.0455, **135.0445**	**+**	**−**	**−**
9	7.28	**Coumaric acid rhamnosyl hexoside**	C_21_H_27_O_12_^–^	471.1503	471.1562	5.95	**163.0401(100)**, 145.0295, 351.1093, **309.0989**, 119.0498	**+**	**−**	**−**
10	8.43	**Di-caffeoylquinic acid**	C_25_H_23_O_12_^–^	515.119	515.1226	3.65	**173.0458(100)**, 179.035, **191.0557**, **353.0885**, 135.0449, 155.0343, 161.0242	**+**	**−**	**−**
11	5.26	**Caffeoylquinic acid hexoside is. I**	C_22_H_27_O_14_^–^	515.1401	515.1414	1.32	**191.0560(100)**, **179.035**, 173.0458, **353.088**, 175.0604, 161.0248, 135.0446	**+**	**+**	**−**
12	6.14	**Caffeoylquinic acid hexoside is. II**	C_22_H_27_O_14_^–^	515.1401	515.1414	1.32	**191.0563(100)**, **179.0349**, 161.0246, 323.0777, **353.0874**, 135.0451	**+**	**+**	**−**
13	6.47	**Caffeoylquinic acid hexoside is. III**	C_22_H_27_O_14_^–^	515.1401	515.1412	1.12	**191.0566(100)**, 515.1442, **179.0355**, 161.0251, 173.0452, 135.0448, **353.0880**, 323.0763, 395.0993	**−**	**+**	**−**
14	6.84	**Caffeoylquinic acid hexoside is. IV**	C_22_H_27_O_14_^–^	515.1401	515.143	2.92	**191.0556(100)**, 323.0776, 161.0255, 134.0382, 341.0850	**−**	**+**	**−**
15	7.03	**Ferulic acid dihexoside**	C_22_H_29_O_14_^–^	517.1557	517.1588	3.07	**175.0401(100)**, **193.0510**, 160.0165, 149.0672, **134.0364**	**−**	**+**	**−**
16	4.45	**Coumaric acid dihexoside + HCOOH**	C_22_H_29_O_15_^–^	533.1506	533.1539	3.25	**163.0401(100)**, 145.0292, **325.0931**, 119.05	**+**	**+**	**−**
***Flavonoids (Flavanon and flavonol glycosides)***										
17	8.85	**Naringenin-7-*O*-hexoside (like Prunin)**	C_21_H_21_O_10_^–^	433.1135	433.1180	4.53	**271.0620(100)**, 151.0041, **119.0498**, 313.0636, 253.0238, 227.0728, 187.0399	**+**	**−**	**−**
18	8.39	**Naringenin-7-*O*-(2”-*O*-rhamnosyl)-hexoside** **(like Naringin)**	C_27_H_31_O_14_^–^	579.1714	579.1708	−0.58	**271.0598(100)**, 151.0034, **119.0560**, 579.1765, **459.1145**, 417.1513	**+**	**−**	**−**
19	8.12	**Kaempferol-3-*O*-(6”-*O*-rhamnosyl)-hexoside**	C_27_H_29_O_15_^–^	593.1506	593.1518	1.15	**285.0401(100)**, 593.1545, 284.0305, 227.0384, 255.0524	**−**	**+**	**−**
20	7.75	**Quercetin-3-*O*-(6”-*O*-rhamnosyl)-hexoside (like Rutin)**	C_27_H_29_O_16_^–^	609.1456	609.1481	2.54	**300.0286(100)**, 609.1499, 301.0349, 271.0247, 255.0328, 178.9997, 151.0049, 343.0454	**−**	**+**	**−**
21	8.17	**Isorhamnetin-3-*O*-(6”-*O*-rhamnosyl)** **-hexoside**	C_28_H_31_O_16_^–^	623.1612	623.163	1.79	**315.0504(100)**, 623.165, 314.0433, **300.0271**, 299.0232, 357.0625	**−**	**+**	**−**
22	9.00	**Laricitrin-3-*O*-[6-*O*-(4-*O*-(*p*-coumaroyl)** **-rhamnosyl)-hexoside]**	C_37_H_37_O_19_^–^	785.1929	785.1956	2.7	**639.1579(100)**, 785.1993, **331.0464**, **315.0148**, 316.0170, 287.0246, 621.1381, 179.0054, 151.0032	**+**	**−**	**−**
***Anthocyanins***										
23	8.02	**Petunidin-3-*O*-(6-*O*-*p*-coumaroyl)** **-rhamnoside-5-*O*-hexoside**	C_37_H_39_O_18_^+^	771.2136	771.2167	3.06	**317.0664(100)**, 771.2166, 479.1191	**+**	**−**	**−**
24	7.62	**Petunidin-3-*O*-[6-*O*-(4-*O*-(*p*-coumaroyl)** **-rhamnosyl)-hexoside]-5-*O*-hexoside**	C_43_H_49_O_23_^+^	933.2665	933.269	2.54	**317.0652(100)**, **771.2156**, 479.1207, 318.0699, 933.2715	**+**	**−**	**−**
25	6.86	**Petunidin-3-*O*-[6-*O*-(4-*O*-(4-*O*-hexosyl)** **-*p*-coumaroyl)-rhamnosyl)-hexoside]-5-*O*-hexoside**	C_49_H_59_O_28_^+^	1095.3193	1095.3201	0.81	**317.0651(100)**, 1095.3234, 479.1185, 455.1599, **933.2677**	**+**	**−**	**−**

Abbreviations: “GMLR”—goat’s milk powder enriched with *L. ruthenicum* extract; “GMLB”—goat’s milk powder enriched with *L. barbarum* extract; “GM”—goat’s milk powder without extract (control sample); “+” identified compounds; and “−“ nonidentified compounds.

**Table 3:** UHPLC Q-ToF MS profile of *Lycium* phenylamides found in methanolic extracts of GM, GMLR, and GMLB powders. Target compounds, expected retention time (RT), molecular formula, calculated mass, exact mass, and MS2 fragments are presented in Table 2.

No	RT	Compounds	Formulas	Calculated Mass	*m*/*z* Exact Mass	mDa	MS Fragments (Main Fragment)	Samples		
								GM LR	GM LB	GM
***Putrescine derivatives***										
26	3.32	***N*-caffeoyl putrescine**	C_13_H_19_N_2_O_3_^+^	251.13960	251.14	0.43	**163.038(100)**, 145.0279, 117.0336	**+**	**−**	**−**
27	3.57	***N*-caffeoyl putrescine-hexoside**	C_19_H_29_N_2_O_8_^+^	413.19240	413.1932	0.81	**163.0389(100)**, 145.0285, 234.1108, 117.0332, **251.1375**	**−**	**+**	**−**
***Non-glycosylated and glycosylated spermidine derivatives***										
28	7.31	***N*-dihydrocaffeoyl-*N′*-coumaroyl spermidine**	C_25_H_34_N_3_O_5_^+^	456.24980	456.2490	−0.85	**222.114(100)**, **147.0445**, 456.2521, 293.1856, 310.2120, 439.2281, 204.1021, **165.0540**, 123.0443, 218.1164	**+**	**−**	**−**
29	7.15	***N,N′*-*bis*-caffeoyl spermidine**	C_25_H_32_N_3_O_6_^+^	470.22910	470.2307	1.59	**220.0969(100)**, **163.0390**, 308.1964, 470.2341, 453.2082, 291.1721, 234.1133, 251.1386, 145.0286	**+**	**−**	**−**
30	6.88	***N*-caffeoyl-*N′*-dihydrocaffeoyl spermidine**	C_25_H_34_N_3_O_6_^+^	472.24480	472.2453	0.54	**220.0988(100)**, **163.0390**, 222.1118, 472.2456, 310.2136, 293.1866, 234.1122, 145.0289, 123.0449, **165.0551**, 455.2234	**+**	**+**	**−**
31	6.74	***N,N′*-*bis*-dihydrocaffeoyl spermidine**	C_25_H_36_N_3_O_6_^+^	474.26040	474.262	1.59	**222.1164(100)**, 474.2772, 236.1279, **165.0548**, 123.0445, 293.1874, 253.1542, 310.2133, 457.2377	**+**	**+**	**−**
32	7.46	***N*-feruloyl-*N′*-dihydrocaffeoyl spermidine**	C_26_H_36_N_3_O_6_^+^	486.26040	486.2602	−0.21	**177.054(100)**, 222.1118, 234.1117, 310.2117, 486.2609, 145.0283, 163.0384, **165.054**, 248.1266, 293.1854, 236.126, 469.2378	**+**	**−**	**−**
33	6.54	***N*-caffeoyl-*N′*-dihydrocaffeoyl spermidine-monohexoside**	C_31_H_44_N_3_O_11_^+^	634.29760	634.2988	1.22	**634.3022(100)**, **222.1118**, **472.249**, **163.0398**, 310.2119, 384.1647, 455.2294, 293.1859, 236.1274, 123.0443, 398.1803, 617.2744	**+**	**+**	**−**
34	6.51	***N,N′*-*bis*-dihydrocaffeoyl spermidine-monohexoside**	C_31_H_46_N_3_O_11_^+^	636.31320	636.3132	−0.03	**636.3222(100)**, **222.1120**, **474.2612**, **165.0535**, 236.1271, 293.1859, 384.1647, 398.1802, 457.2364, 619.2884	**+**	**−**	**−**
35	6.72	***N,N′*-*bis*-caffeoyl spermidine-dihexoside**	C_37_H_52_N_3_O_16_^+^	794.33480	794.3370	2.24	**796.3488(100)**, **794.3370**, **632.2817**, 470.2479, 382.1479, 220.0950, 163.0375	**−**	**+**	**−**
36	6.41	***N*-caffeoyl-*N′*-dihydrocaffeoyl spermidine-dihexoside**	C_37_H_54_N_3_O_16_^+^	796.35040	796.3523	1.89	**796.3527(100)**, **634.2976**, **472.2497**, 384.1640, 310.2118, **222.1113**, 163.0385, **220.0959**, 382.1500	**+**	**+**	**−**
37	6.34	***N,N′*-*bis*-dihydrocaffeoyl spermidine-dihexoside**	C_37_H_56_N_3_O_16_^+^	798.36610	798.3661	0.04	**798.3681(100)**, 636.3119, **474.2593**, **384.164**, **222.1115**	**+**	**+**	**−**
38	6.52	***N*-caffeoyl-*N′*-dihydrocaffeoyl spermidine-trihexoside**	C_43_H_64_N_3_O_21_^+^	958.40320	958.4039	0.67	**958.4058(100)**, **796.3462**, **634.2952, 472.2510**, 544.1995, 310.2113, 220.0961, **163.0386**	**−**	**+**	**−**
***Non-glycosylated and glycosylated spermine derivatives***										
39	6.25	***N*-caffeoyl-*N′*-dihydrocaffeoyl spermine**	C_28_H_41_N_4_O_6_^+^	529.30260	529.3036	0.99	**220.0957(100)**, **293.1855**, **291.1705**, **222.1116**, 367.2694, 163.0389, 129.1388, 511.2911, 165.0538, 512.2833, 529.3044	**+**	**−**	**−**
40	5.99	***N,N′*-*bis*-dihydrocaffeoyl spermine (kukoamines)**	C_28_H_43_N_4_O_6_^+^	531.31830	531.3185	0.24	**293.1863(100)**, **222.1124**, 531.3253, 165.0549, 123.0449, **310.2116**, 367.2700, 514.2978	**+**	**−**	**−**
41	5.91	***N*-caffeoyl-*N′*-dihydrocaffeoyl spermine-monohexoside**	C_34_H_51_N_4_O_11_^+^	691.35540	691.3558	0.37	**693.3715(100)**, 293.1868, 455.2387, **222.1120**, **220.096**, 384.1635, 531.3168, **529.3107**, 291.1698, 163.0391, 675.3593	**+**	**−**	**−**
42	5.89	***N,N′*-*bis*-dihydrocaffeoyl spermine-monohexoside**	C_34_H_53_N_4_O_11_^+^	693.37110	693.3718	0.72	**693.3734(100)**, 293.1866, 455.2386, **222.1124**, **531.3169**, 676.3508, 384.1649, 165.0537	**+**	**−**	**−**
43	5.84	***N,N′*-*bis*-dihydrocaffeoyl spermine-dihexoside**	C_40_H_63_N_4_O_16_^+^	855.42390	855.4248	0.89	**855.4283(100)**, **455.2404**, **693.3706**, 384.1647, 293.1850, 222.1120	**−**	**+**	**−**

Abbreviations: “GMLR”—goat’s milk powder enriched with *L. ruthenicum* extract; “GMLB”—goat’s milk powder enriched with *L. barbarum* extract; “GM”—goat’s milk powder without extract (control sample); “**+**” identified compounds; and “**−**“ nonidentified compounds.

**Table 4:** Total phenolic content, total protein content, and antioxidant properties of goat′s milk and goat′s milk/goji extract powders.

Parameter	Unit	GMLR	GMLB	GM
TPC	mg GAE/100 mL	6.63 ± 0.16 ^a^	4.77 ± 0.0001 ^c^	5.41 ± 0.09 ^b^
TPrC	mg BSA/100 mL	27.85 ± 3.20 ^b^	26.00 ± 1.44 ^b^	35.44 ± 2.74 ^a^
FRP	µg AA/mL	13.98 ± 1.37	/	/
ABTS	µg TE/mL	81.97 ± 4.54 ^a^	24.017 ± 2.42 ^b^	8.33 ± 1.66 ^c^
FCC	µg EDTA/mL	331.10 ± 2.34 ^b^	321.51 ± 5.38 ^b^	396.92 ± 1.10 ^a^

Abbreviations: “GMLR”—goat’s milk powder enriched with *L. ruthenicum* extract; “GMLB”—goat’s milk powder enriched with *L. barbarum* extract; “GM”—goat’s milk powder without extract (control sample); “TPC”—total phenolic content; “TPrC”—total protein content; “FCC”—ferrous ion-chelating capacity; “ABTS”—ABTS^•+^ radical scavenging activity; “FRP”—ferric ion-reducing power; “GAE”—gallic acid; “BSA”—bovine serum albumin; “AA”—ascorbic acid; “TE”—trolox and “EDTA ” ethylenediaminetetraacetic acid; “/”—not determined. Values are presented as mean ± standard deviation (mean ± SD). Different letters in the same row denote a significant difference (*p* < 0.05), according to *Tukey’s* test.

**Table 5:** The results of the prebiotic activity of GM, GMLR, and GMLB on the growth of probiotic strains of microorganisms.

Goat′s Milk Powder and Goat′s Milk/Goji Extract Powders	Concentration				
	5 mg/mL	2.5 mg/mL	1.25 mg/mL	0.625 mg/mL	0.312 mg/mL
GMLB					
*Lactobacillus plantarum*	157.9 ± 18.9 ^ab^*	141.4 ± 17.0 ^ab^*	132.9 ± 15.9 ^ab^*	122.0 ± 14.6 ^ab^*	109.5 ± 8.8 ^ab^*
*Lactobacillus reuteri*	132.1 ± 15.8 ^a^*	127.7 ± 15.3 ^ab^*	120.8 ± 14.5 ^ab^*	116.4 ± 14.0 ^ab^*	107.5 ± 8.6 ^ab^*
*Lactobacillus rhamnosus*	141.4 ± 17.0 ^ab^*	130.9 ± 15.7 ^ab^*	114.8 ± 13.8 ^ab^*	110.5 ± 13.3 ^ab^*	107.9 ± 8.6 ^ab^
*Streptococcus thermophilus*	151.6 ± 18.2 ^ab^*	129.7 ± 15.6 ^ab^*	119.4 ± 14.3 ^ab^*	112.9 ± 13.5 ^ab^*	105.2 ± 8.4 ^ab^
*Saccharomyces boulardii*	169.9 ± 20.4 ^ab^*	157.1 ± 18.8 ^ab^*	141.0 ± 16.9 ^ab^*	128.8 ± 15.5 ^ab^	115.4 ± 9.2 ^ab^
*Lactobacilli/Bifidobacterium mixture*	168.2 ± 20.2 ^ab^*	161.2 ± 19.3 ^ab^*	146.3 ± 17.6 ^ab^*	133.2 ± 16.0 ^ab^	118.9 ± 9.5 ^ab^*
GMLR					
*Lactobacillus plantarum*	184.5 ± 22.1 ^ac^*	172.0 ± 20.6 ^ac^	163.8 ± 19.7 ^ac^*	148.4 ± 17.8 ^ac^	125.3 ± 10.0 ^ac^*
*Lactobacillus reuteri*	162.9 ± 19.5 ^ac^*	147.8 ± 17.7 ^ac^	136.2 ± 16.3 ^ac^*	128.9 ± 15.5 ^ac^	113.2 ± 9.1 ^ac^*
*Lactobacillus rhamnosus*	180.9 ± 21.7 ^ac^*	159.2 ± 19.1 ^ac^*	130.9 ± 15.7 ^ac^*	121.7 ± 14.6 ^ac^*	114.5 ± 9.2 ^ac^
*Streptococcus thermophilus*	170.3 ± 20.4 ^ac^*	152.9 ± 18.3 ^ac^*	137.4 ± 16.5 ^ac^	125.8 ± 15.1 ^ac^	113.5 ± 9.1 ^ac^*
*Saccharomyces boulardii*	211.5 ± 25.4 ^ac^*	179.5 ± 21.5 ^ac^*	156.1 ± 18.7 ^ac^*	144.6 ± 17.3 ^ac^*	130.8 ± 10.5 ^ac^*
*Lactobacilli/Bifidobacterium mixture*	193.9 ± 23.3 ^ac^*	181.8 ± 21.8 ^ac^*	165.9 ± 19.9 ^ac^*	152.3 ± 18.3 ^ac^	133.2 ± 10.7 ^ac^*
GM					
*Lactobacillus plantarum*	122.4 ± 14.7 ^bc^*	109.2 ± 13.1 ^bc^*	104.6 ± 12.6 ^bc^*	102.6 ± 12.3 ^bc^*	100.7 ± 8.1 **^bc^**
*Lactobacillus reuteri*	117.0 ± 14.0 ^c^*	110.7 ± 13.3 ^bc^*	105.0 ± 12.5 ^bc^*	100.6 ± 12.1 ^bc^*	100.0 ± 8.0 **^bc^**
*Lactobacillus rhamnosus*	119.7 ± 14.4 ^bc^*	109.2 ± 13.1 ^bc^*	104.6 ± 12.6 **^bc^**	102.0 ± 12.2 **^bc^**	101.3 ± 8.1 **^bc^**
*Streptococcus thermophilus*	121.3 ± 14.6 ^bc^*	111.6 ± 13.4 ^bc*^	105.2 ± 12.4 **^bc^**	101.9 ± 12.2 **^bc^**	103.2 ± 8.3 **^bc^**
*Saccharomyces boulardii*	123.1 ± 14.8 ^bc^*	113.5 ± 13.6 ^bc^*	107.1 ± 12.8 ^bc^*	103.2 ± 12.4 **^bc^**	101.9 ± 8.2 **^bc^**
*Lactobacilli/Bifidobacterium mixture*	136.0 ± 16.3 ^bc^*	126.6 ± 15.2 ^bc^*	118.7 ± 14.2 **^bc^**	112.6 ± 13.5 **^bc^**	106.5 ± 8.5 **^bc^**

The results are presented as the mean percentage of growth stimulation ± SD of probiotic strains after cultivation with goji/milk powders in the range of 0.312–5 mg/mL. Letters a–c indicate significant differences (*p* < 0.05) between the growths of probiotics strain(s) treated with the same concentration of different extract/milk combinations or goat milk powder (a-GMLB/GMLR; b-GMLB/GM; and c-GMLR/GM), and * indicate significant differences (*p* < 0.05) between the growths of probiotic strain(s) treated with the increasing concentrations of the same extract. Abbreviations: “GMLR”—goat’s milk powder enriched with *L. ruthenicum* extract; “GMLB”—goat’s milk powder enriched with *L. barbarum* extract; and “GM”—goat’s milk powder without extract (control sample).

## Data Availability

The original contributions presented in this study are included in the article. Further inquiries can be directed to the corresponding authors.
